# Inactivation of hnRNP K by Expanded Intronic AUUCU Repeat Induces Apoptosis Via Translocation of PKCδ to Mitochondria in Spinocerebellar Ataxia 10

**DOI:** 10.1371/journal.pgen.1000984

**Published:** 2010-06-10

**Authors:** Misti C. White, Rui Gao, Weidong Xu, Santi M. Mandal, Jung G. Lim, Tapas K. Hazra, Maki Wakamiya, Sharon F. Edwards, Salmo Raskin, Hélio A. G. Teive, Huda Y. Zoghbi, Partha S. Sarkar, Tetsuo Ashizawa

**Affiliations:** 1 Department of Neurology, University of Texas Medical Branch, Galveston, Texas, United States of America; 2 Department of Internal Medicine, University of Texas Medical Branch, Galveston, Texas, United States of America; 3 Center for Health and Biological Sciences, Pontifícia Universidade Católica do Paraná, Curitiba, Brazil; 4 Neurology Service, Federal University of Paraná, Curitiba, Brazil; 5 Howard Hughes Medical Institute, Baylor College of Medicine, Houston, Texas, United States of America; 6 Department of Neurology, University of Florida, Gainesville, Florida, United States of America; The Hospital for Sick Children and University of Toronto, Canada

## Abstract

We have identified a large expansion of an ATTCT repeat within intron 9 of *ATXN10* on chromosome 22q13.31 as the genetic mutation of spinocerebellar ataxia type 10 (SCA10). Our subsequent studies indicated that neither a gain nor a loss of function of ataxin 10 is likely the major pathogenic mechanism of SCA10. Here, using SCA10 cells, and transfected cells and transgenic mouse brain expressing expanded intronic AUUCU repeats as disease models, we show evidence for a key pathogenic molecular mechanism of SCA10. First, we studied the fate of the mutant repeat RNA by *in situ* hybridization. A Cy3-(AGAAU)_10_ riboprobe detected expanded AUUCU repeats aggregated in foci in SCA10 cells. Pull-down and co-immunoprecipitation data suggested that expanded AUUCU repeats within the spliced intronic sequence strongly bind to hnRNP K. Co-localization of hnRNP K and the AUUCU repeat aggregates in the transgenic mouse brain and transfected cells confirmed this interaction. To examine the impact of this interaction on hnRNP K function, we performed RT–PCR analysis of a splicing-regulatory target of hnRNP K, and found diminished hnRNP K activity in SCA10 cells. Cells expressing expanded AUUCU repeats underwent apoptosis, which accompanied massive translocation of PKCδ to mitochondria and activation of caspase 3. Importantly, siRNA–mediated hnRNP K deficiency also caused the same apoptotic event in otherwise normal cells, and over-expression of hnRNP K rescued cells expressing expanded AUUCU repeats from apoptosis, suggesting that the loss of function of hnRNP K plays a key role in cell death of SCA10. These results suggest that the expanded AUUCU–repeat in the intronic RNA undergoes normal transcription and splicing, but causes apoptosis via an activation cascade involving a loss of hnRNP K activities, massive translocation of PKCδ to mitochondria, and caspase 3 activation.

## Author Summary

In an earlier study, we showed that the mutation of spinocerebellar ataxia 10 (SCA10) is an enormous expansion of a gene segment, which contains a tandemly repeated 5-base (ATTCT) unit. Since SCA10 is the only known human disease that is proven to be caused by 5-base repeat expansion, it is important to learn how this novel class of mutation causes the disease. We found that the mutation produces an expanded RNA repeat, which aberrantly accumulates in SCA10 cells and interacts with a major RNA–binding protein. When we expressed expanded RNA repeats or decreased the RNA–binding protein level in cultured cells, either of these manipulations produced a specific type of cell death that is associated with a massive transfer of a key enzyme called protein kinase C delta to mitochondria. We also showed that either blocking the expanded AUUCU repeat or replenishing hnRNP K rescues cells from the cell death induced by the SCA10 mutation. Together, we conclude that the mutant RNA inactivates hnRNP K and kills cells by triggering the specific cell-death mechanism. Our data provide important clues for therapeutic intervention in SCA10.

## Introduction

Spinocerebellar ataxia type 10 (SCA10) is an autosomal dominant neurodegenerative disease presented with progressive pancerebellar ataxia, leading to total disability [1]–[4]. Approximately 60% of the SCA10 patients also suffer from epilepsy with complex partial seizures and generalized tonic-clonic seizures, which become life-threatening due to development of status epilepticus [3]–[5]. The disease-causing genetic mutation is a large (up to 22.5 kb) expansion of a pentanucleotide, ATTCT, repeat present within the ninth intron of the *ATXN10* gene on chromosome 22q13.31 [6].

In the last two decades, investigators identified a group of diseases caused by expansions of short tandem repeats, also known as microsatellite repeats. Most of these mutations involve unstable trinucleotide repeats located in different regions of respective genes. The roles of repeat expansion mutations in the pathogenic mechanism of these diseases are diverse and complex [7], [8]. However, in a simplistic view an expanded repeat in the coding region produces an elongated tract of repetitive amino acid residues with a gain of toxic function at the protein level, whereas a triplet repeat expansion in 5′- and 3′-untranslated regions (UTR) may result in an altered transcription level of the gene or a production of toxic RNA transcript containing expanded ribonucleotide triplets. Friedreich's ataxia (FRDA) is the only known disease caused by an expansion of an intronic trinucleotide repeat. Typical FRDA mutations are large GAA repeats located in intron 1 of the *FXN* gene, which severely hinders the transcription of the *FXN* gene, leading to the autosomal recessive phenotype [9].

SCA10 and myotonic dystrophy type 2 (DM2) are only human diseases caused by non-trinucleotide microsatellite expansion mutations although an insertion of a large pentanucleotide repeat has recently been reported to be associated with SCA31 [10]. In DM2 the mutation is a large (up to 44 kb) expansion of CCTG tetranucleotide repeat in intron 1 of the *ZNF9* gene. Thus, it is an interesting coincidence that non-trinucleotide mutations in DM2 and SCA10 are both large expansions located in an intron and causing autosomal dominant phenotypes. In DM2, expanded CCUG tetranucleotide repeat transcripts accumulate mostly in nuclear foci, and sequestrate the muscleblind like 1 (MBNL1) protein into the RNA foci [11], [12]. The resultant depletion of MBNL1 causes splicing dysregulation of a variety of RNA transcritpts similar to DM1. Splicing misregulation is thought to be the primary pathogenic mechanism in DM1 and DM2.

In SCA10 the number of ATTCT repeats ranges from 10 to 29 in normal individuals, and increases up to 4,500 in patients [13], [14]. The *ATXN10* gene consists of 12 exons spanning 172.8 kb, and encodes ataxin 10, which contains two armadillo repeats known to mediate protein-protein interaction. Knock-down of *ATXN10* by RNAi induces cell death in primary cerebellar neurons [15], whereas over-expression of *ATXN10* activates the mitogen-activated protein kinase cascade and promotes neurite extension in PC12 cells [16]. While *ATXN10* is expressed in a wide variety of tissues, expression is especially strong in brain, heart and muscle. Although these data suggest that ataxin 10 plays a role in neuronal survival and differentiation, the exact function of ataxin 10 remains unknown. Thus, it is plausible that a large expansion of the ATTCT repeat may interfere with the transcription, like the GAA repeat expansion does in FRDA, leading to a loss of function of ataxin 10. However, we recently demonstrated that neither a gain nor a loss of the function of *ATXN10* is the primary pathogenic mechanism of SCA10 [17]. Analyses of SCA10 fibroblasts showed that the *ATXN10* mRNA levels remain unaltered in spite of the repeat expansion [6], [17]. In addition, transcription of the mutant alleles and post-transcriptional splicing of the mutant *ATXN10* transcript remain largely unaltered in SCA10 patients [17]. Furthermore, homozygous *Atxn10* knockout (*Atxn10*−/−) mice showed embryonic lethality while heterozygous (Atxn10+/−) mice showed no phenotype [17]. Finally, a recent report described patients with balanced translocation t(2;22)(p25.3:q13.31), in which the breakpoint of chromosome 22q13.31 disrupted intron 2 of *ATXN10* [18]. These patients were totally asymptomatic, suggesting that haploinsufficiency of *ATXN10* does not cause SCA10.

In the present study, we examine whether the expanded AUUCU RNA repeat in the mutant *ATXN10* transcript is the principal pathogenic molecule capable of triggering neuronal death in SCA10. We demonstrate that the expanded AUUCU repeat within the spliced intron interacts with hnRNP K, and this RNA-protein interaction results in loss of hnRNP K function, translocation of Protein Kinase C δ (PKCδ) to mitochondria and activation of apoptosis in SCA10 cells. Furthermore, we observe that targeted inactivation of the mutant *ATXN10* transcripts in SCA10 cells significantly reduces mitochondrial translocation of PKCδ. Together, these results define a key pathogenic mechanism of SCA10 and provide clues for potential therapeutic strategies.

## Results

### Mutant *ATXN10* transcripts are accumulated as inclusion aggregates in SCA10

**Figure 1:**
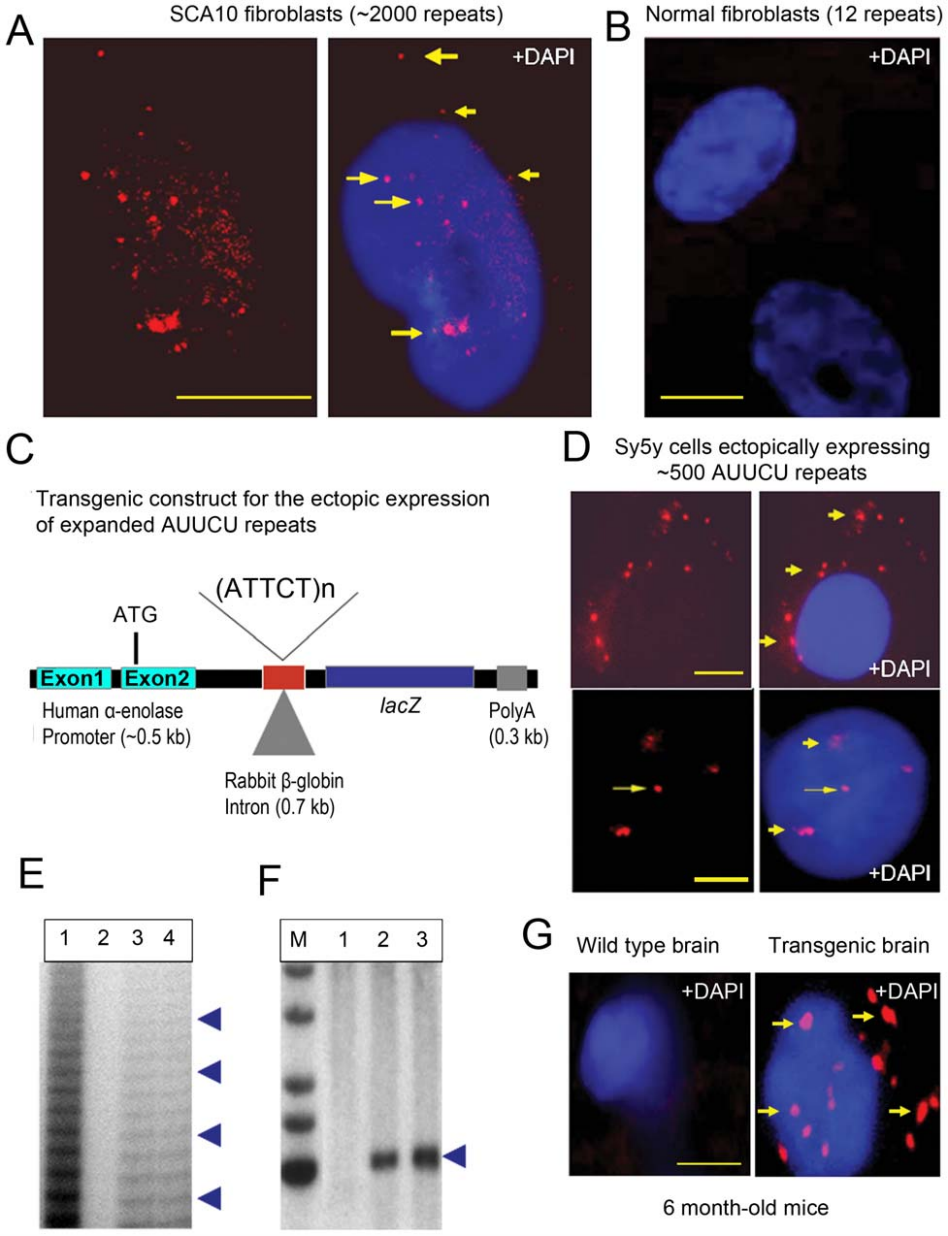
Expanded AUUCU transcripts form discrete aggregates. (A) *FISH* on SCA10 fibroblasts encoding ∼2,000 ATTCT repeats showing multiple, bright RNA aggregates (arrow) in the nuclei, nuclear boundary and cytoplasm. (B) *FISH* on normal control fibroblasts hybridized with a Cy3-labeled (AGAAU)_10_ probe. (C) Schematic drawing of the transgenic constructs including a human *α-enolase* promoter, 2^nd^ intron of rabbit *β-globin* gene, *LacZ* gene, expanded ∼500 ATTCT repeats cloned within the intron (or normal range of 12 repeats for control), and Bovine Growth Hormone (BGH) polyA sequences. (D) *FISH* on Sy5y cells ectopically expressing intronic ∼500 AUUCU repeats from the transgenic constructs shown in Figure 1C. Figure showing the nuclear, perinuclear as well as cytoplasmic foci (arrow). (E) Ladder-PCR analysis of mouse genomic DNA showing the presence of expanded ATTCT repeats, lane 1: Positive control from SCA10 patients; Lane 2: Transgenic mouse containing 12 ATTCT repeats, lane 3 & 4: Transgenic mouse encoding ∼500 ATTCT repeats. (F) Southern blot of the *Xba*I-*Hin*dII digested genomic DNA from the transgenic F1 encoding ∼500 ATTCT repeats (lane 2, 3). Lane 1: Wild-type control mouse DNA. Lane 2 and 3: Transgenic mice expressing ∼500 ATTCT repeats. Southern blot shows the presence of ∼500 ATTCT repeats. A DNA fragment (∼500 bp) of the 5′ of the intron 9 of *ATXN10* gene was used as the Southern probe. (G) *FISH* on sagittal sections of the wild type (left panel) and transgenic mouse brain encoding ∼500 AUUCU repeats (right panel). Bars represent 10 mm in this and all other figures.

We propose that the mutant *ATXN10* transcripts containing expanded AUUCU repeats contribute towards the SCA10 phenotype. To investigate whether the sub-cellular distribution and fate of the mutant *ATXN10* transcripts are altered, RNA *FISH* analysis with a Cy3-(AGAAU)_10_ riboprobe was performed on SCA10 fibroblasts containing ∼2000 or ∼1,000 ATTCT repeats and on normal fibroblasts expressing the wild type *ATXN10* transcripts containing 12 AUUCU repeats. The (AGAAU)_10_ riboprobe detected the presence of nuclear and cytoplasmic aggregates in SCA10 fibroblasts (Figure 1A; arrows, also Figure S1A; arrow), but not in normal fibroblasts (Figure 1B). These aggregates observed in this and other Figures were resistant to DNAse and disappear after RNAse treatment. Since our previous study showed that the 9^th^ intron of the *ATXN10* gene (66,421 bp) encoding the expanded AUUCU repeats is spliced normally [17], our present results imply that the intron 9 sequences are spliced and partly translocated to the cytoplasm in SCA10 fibroblasts. *FISH* with an anti-sense probe specific for exon 9 of the *ATXN10* gene showed no significant binding in the same SCA10 fibroblasts (data not shown), confirming that the aggregated AUUCU repeat sequences are spliced from the mutant *ATXN10* transcripts. These findings suggest that intron 9 containing the expanded AUUCU repeat is spliced out of the mutant *ATXN10* transcripts, but expanded AUUCU repeats within the spliced intron 9 are resistant to degradation, and deposited as aggregates in nuclei and in cytoplasm in SCA10 cells.

### Ectopically expressed AUUCU repeats are accumulated as distinct aggregates

We determined whether expanded AUUCU repeats alone are sufficient to form aggregates. Untranslated ∼500 AUUCU repeats from a transgene (Figure 1C) were expressed in human neuroblastoma Sy5y cells. The transgene is designed to express an expanded ATTCT repeat within the rabbit *β-globin* intron downstream of the human *α-enolase* promoter and upstream of the *LacZ* reporter. Using RT-PCR analysis, we confirmed that the AUUCU-repeat-containing the rabbit *β-globin* intron is spliced from the transcript when the transgene is expressed in Sy5y cells (data not shown). *FISH* analysis of the Sy5y cells expressing ∼500 AUUCU repeats showed SCA10-like nuclear and cytoplasmic aggregates (Figure 1D). However, under identical conditions, aggregates were not detected in Sy5y cells expressing the *lacZ* transcripts encoding shorter repeats (12 or 25 repeats) (data not shown). *FISH* analysis of transfected normal human fibroblasts expressing ∼500 AUUCU repeats also showed SCA10-like nuclear and cytoplasmic aggregates (Figure S1B; arrow). These data indicate that even when the expanded AUUCU repeat is ectopically expressed, the intronic sequence is spliced from the transcript of the transgene, becomes resistant to degradation, and aggregates in nuclear and cytoplasmic foci in Sy5y cells.

We also determined whether transcripts with expanded AUUCU sequences form similar aggregates in mouse brain. Transgenic mouse lines using the construct described in Figure 1C were generated. Repeat-primed PCR (RP-PCR) analyses of genomic DNA from these mice showed the presence of expanded ATTCT repeats (Figure 1E), and Southern analyses confirmed the presence and integrity of ∼500 ATTCT repeats (Figure 1F). The (AGAAU)_10_ riboprobe detected distinct intracellular aggregates in brains from 6-month-old (Figure 1G) and 3-month-old (Figure S1C) transgenic mice, but not in control mouse brains. Importantly, similar to the SCA10 cells, we observed a large number of aggregates not only in the nucleus but also in the cytoplasm, and they were more abundant in 6-month-old than 3-month-old mice (Figure 1G and Figure S1C). The formation of SCA10-like aggregates in these cells and transgenic mouse brains confirms that the expanded AUUCU repeats are necessary and sufficient to form nuclear and cytoplasmic aggregates. Moreover, these large foci suggest that the expanded AUUCU-RNA repeats may aggregate as insoluble RNA-protein complexes, as described in other repeat expansion disorders [7], [8].

### Expanded AUUCU transcripts activate apoptosis

**Figure 2:**
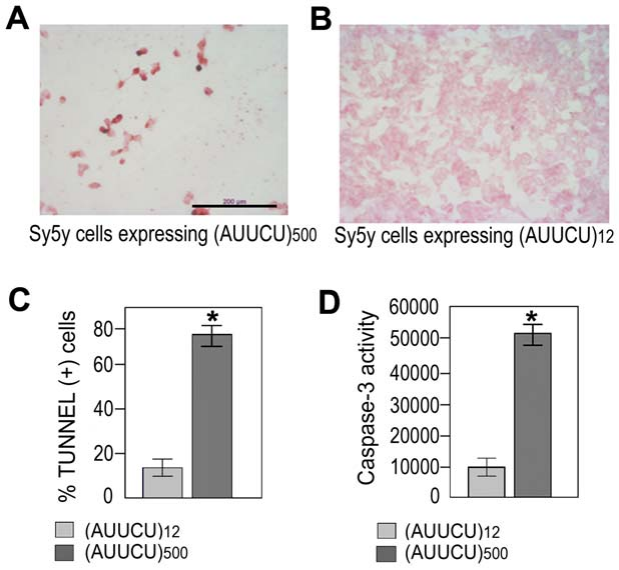
Expanded AUUCU repeat induces apoptosis. (A) TUNEL assay on Sy5y cells expressing ∼500 intronic AUUCU repeats. (B) TUNEL assay on Sy5y cells expressing 12 intronic AUUCU repeats. (C) Percentage of cells undergoing apoptosis when 12 and ∼500 intronic AUUCU repeats are expressed (n = 6; p<0.0002). (D) Bar diagram showing Caspase-3 activities as relative fluorescent units (RFU) when Sy5y cells express 12 and ∼500 AUUCU intronic repeats (right bars) repeat (*n = 3, p<0.0001).

Light-microscopic analysis of the Sy5y cells expressing the ∼500 AUUCU repeats showed a dramatic increase in cell death (Figure 2A), whereas cells expressing normal-size repeats showed virtually no cell death (Figure 2B). A TUNEL assay revealed that more than 70% of cells expressing the ∼500 AUUCU repeats underwent apoptosis 48 hours after transfection (Figure 2A and 2C), while cells expressing 12 AUUCU repeats did not undergo apoptosis (Figure 2B and 2C) (p<0.0001). Furthermore, caspase-3 activity was significantly higher in cells expressing ∼500 AUUCU repeats than in control cells (p<0.0001) (Figure 2D), suggesting that the expanded AUUCU repeats activate caspase-3-mediated apoptosis. We also observed that expression of ∼500 AUUCU repeats cause apoptosis in PC12 cells (Figure S2).

### Expanded AUUCU RNA binds to heteronuclear ribonuclear protein K (hnRNP K)

**Figure 3:**
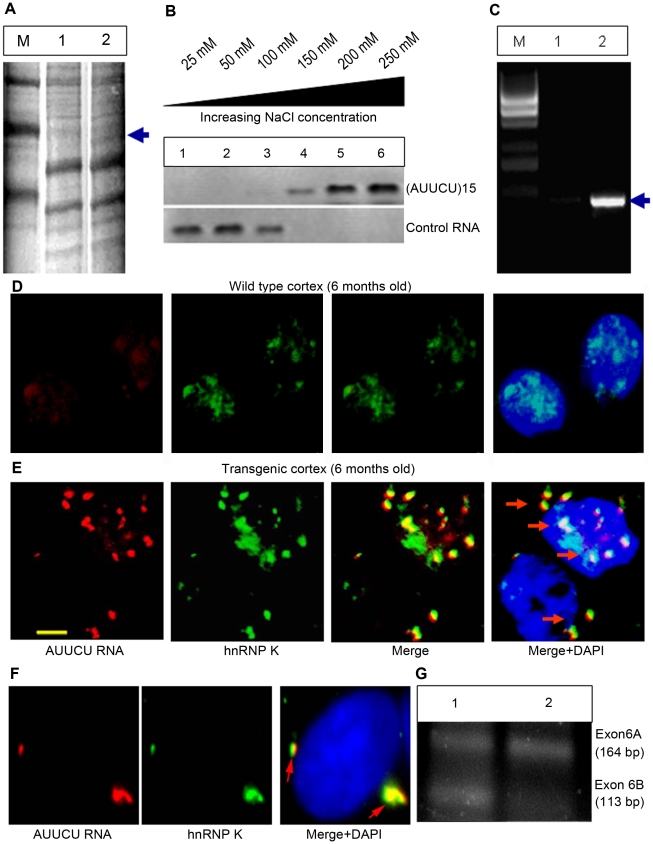
Expanded AUUCU-RNA binds to hnRNP K. (A) PAGE showing the proteins pulled down from mouse brain extract with biotin-labeled AUUCU-RNA. Lane 1: Molecular weight marker, Lane 2: Proteins bound to *lacZ* transcripts with 12 AUUCU repeats; Lane 3: Proteins bound to ∼500 AUUCU repeats + lacZ transcripts. The unique protein band that was analyzed by mass spectrometry is marked with arrow. (B) Western blot analysis of hnRNP K extracted from the RNA-protein complex at increasing salt concentration. Purified hnRNP K was incubated with single-stranded (AUUCU)_15_ RNA or control RNA {(UUUCC)_3_(CCCUU)_3_(UUUUC)_3}_ before extraction. The top blot is hnRNP K extracted from (AUUCU)_15_ RNA-protein complex and the bottom blot is the hnRNP K fractions extracted from hnRNP K-control RNA complex. (C) RT-PCR analysis of the RNA-Co-IP pellet from normal (lane 1) and SCA10 fibroblast extract (lane 2). The pulled down IP from SCA10 fibroblast extract shows the presence of intron 9 sequence of *ATXN10* gene. M = 1 kb DNA ladder. (D) *FISH* on the sagittal section of the wild type control mouse brain. (E) *FISH* showing co-localization of endogenous hnRNP K (green) with AUUCU RNA (red) in sagittal sections of the SCA10 transgenic mouse brain expressing ∼500 intronic AUUCU repeats. (F) *FISH* on Sy5y cells showing co-localization of hnRNP K with expanded AUUCU repeats. Red fluorescence: AUUCU RNA; Green fluorescence: GFP-tagged hnRNP K and Yellow/Orange fluorescence: Overlap of red and green fluorescence (arrow). (G) RT-PCR analysis of total RNA from normal (lane 1) and SCA10 (lane 2) fibroblasts showing aberrant splicing of *β-tropomyosin.*

The distinct aggregates in SCA10 cells and transgenic mouse brains led us to hypothesize that the expanded AUUCU repeat RNA may interact with proteins, and that such interactions may have pathogenic significance. We pulled down proteins from mouse brain extracts using biotin-labeled expanded AUUCU RNA repeats and analyzed them by SDS-PAGE (Figure 3A). The unique protein that was reproducibly and repeatedly pulled down (n = 6) was identified by mass spectrometry as hnRNP K (Figure 3A; arrow). hnRNP K contains three K-homology (KH) domains that mediate its interactions with RNA and a K interactive (KI) region with proline-rich docking sites important for src homology domain binding. To establish the specificity of the interaction of hnRNP K with AUUCU RNA, purified hnRNP K was incubated with single-stranded (AUUCU)_15_ RNA and extracted with buffers containing increasing salt concentrations. The data show a significant affinity of hnRNP K with (AUUCU)_15_ RNA even in the presence of 250 mM NaCl; in contrast hnRNP K is completely dissociated from the control RNA at significantly lower (≤100 mM) salt concentrations (Figure 3B). Thus, hnRNP K can bind tightly to AUUCU repeats, in addition to the consensus sequence, U(C3-4)U/A [19]. We next immuno-precipitated (IP) hnRNP K from SCA10 and normal fibroblasts and determined the presence of intron 9 of the *ATNX10* transcript in the IP pellets. RT-PCR analysis of the IP pellets showed the presence of the intron 9 sequence of the *ATXN10* transcript when hnRNP K was precipitated from SCA10 fibroblasts but not from normal fibroblasts (Figure 3C). We did not detect the presence of exon 9 or exon 10 in the pellets, further corroborating the idea that intron 9 containing the expanded AUUCU repeat is spliced from the *ATXN10* transcript. These data indicate that hnRNP K is tightly associated with the expanded AUUCU repeat within the intron 9 sequence in SCA10 cells.

To demonstrate the *in vivo* interaction of the expanded repeat with hnRNP K, we investigated the co-localization of hnRNP K with AUUCU RNA in transgenic mouse brain. The expanded AUUCU repeat aggregates were visualized by *FISH*, and hnRNP K was detected with anti-hnRNP K antibody by immunofluorescence. Sagittal sections of hippocampus CA1 (Figure 3E) and cerebral cortex (Figure S3) from the 6-month-old transgenic mice showed distinct co-localization of ∼500 AUUCU aggregates with endogenous hnRNP K. In contrast, control mouse brains showed no foci (Figure 3D). We next transfected Sy5y cells with two plasmids: one to express the ∼500 AUUCU repeat (Figure 1C) and the other to express GFP-tagged hnRNP K. *FISH* analysis of the double-transfected cells revealed significant co-localization of the red fluorescence from the AUUCU RNA repeat and the green fluorescence from the GFP-hnRNP K (Figure 3F; arrow), indicating that hnRNP K exists as a RNA-protein complex with AUUCU RNA *in vivo*.

We assessed whether binding of hnRNP K with the expanded AUUCU RNA interferes with hnRNP K activity by studying hnRNP K-regulated alternative splicing of transcripts. hnRNP K is known to regulate alternative splicing of exon 6A and 6B, the mutually exclusive exons of the *β-tropomyosin* gene in vertebrates, and decreased hnRNP K activity has been shown to increase the inclusion of exon 6A and the exclusion of exon 6B [20], [21]. RT-PCR analysis showed that exon 6A is predominantly included in the mature *β-tropomyosin* transcripts in SCA10 cells compared to normal control cells (Figure 3G), suggesting that the hnRNP K activity is decreased in SCA10 cells. Consistent with these results, splicing of β-tropomyosin was also markedly altered in normal fibroblasts ectopically expressing expanded AUUCU repeats (data not shown).

### Down-regulation of hnRNP K activates apoptosis

**Figure 4:**
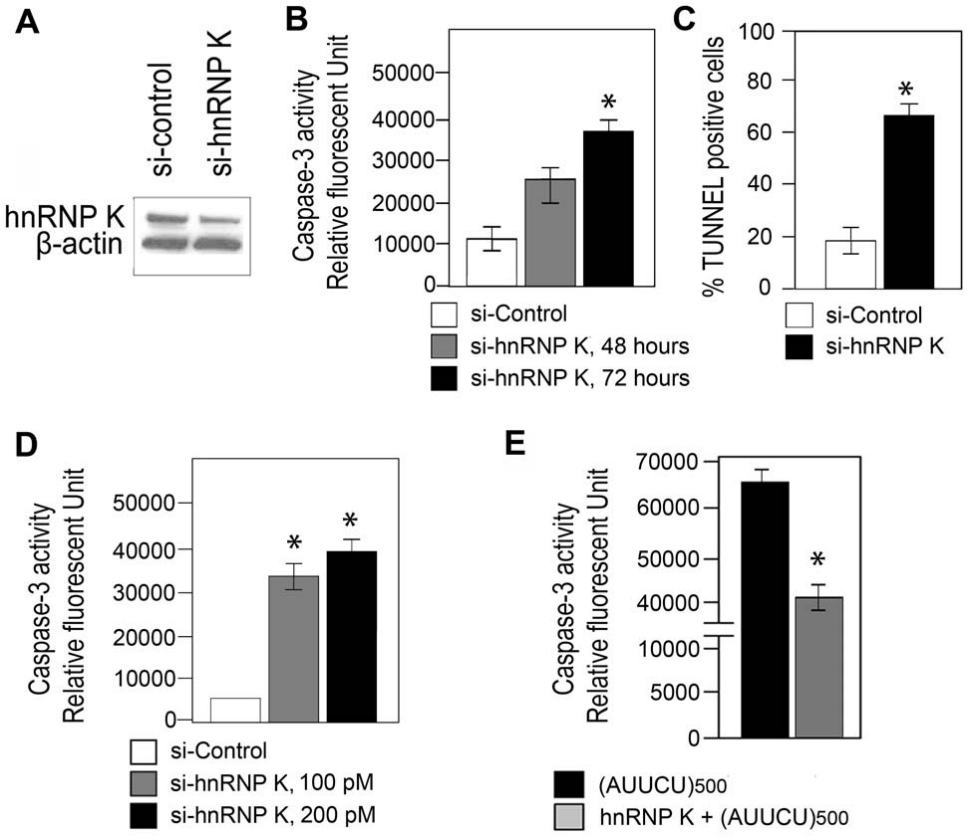
Targeted inactivation of *hnRNP K* triggers apoptosis whereas ectopic expression of hnRNP K rescues AUUCU–mediated apoptosis. (A) Western showing hnRNP K levels in Sy5y cells 72 hours after trasfecting with control siRNA and with 100pmoles siRNA for *hnRNP K*. *β-actin* control is shown. (B) Caspase-3 activity of Sy5y cells is shown as the relative fluorescent units, 48 and 72 hours after treatment with 100pmoles si*hnRNP K* (*n = 3, p<0.0001) and 72 hours after treatment with control siRNA. (C) TUNEL assay showing the percentage of Sy5y cells undergoing apoptosis after treating with control and si*hnRNP K* (*n = 6, p<0.0001). (D) Caspase-3 activities of Sy5y cells is shown as the relative fluorescent units (RFU), after treating with either 100 pM (*n = 3, p = 0.0001) or 200pmoles si*hnRNP K* compared to 100pmoles control siRNA. (E) Caspase-3 activities of Sy5y cells expressing ∼500 AUUCU repeats (black); Sy5y cells expressing hnRNP K and ∼500 AUUCU repeats (grey).

To understand the possible pathogenicity of a loss of hnRNP K function in SCA10, we treated Sy5y cells with four different hnRNP K siRNA duplexes. The sequence that most significantly and reproducibly decreased hnRNP K protein level was used at titrating concentrations to knockdown hnRNP K in Sy5y cells. Western blot analysis showed that cells treated with 100 pM hnRNP K siRNA had >50% reduction in hnRNP K protein level, compared to that in cells treated with control siRNA (Figure 4A). We detected no significant cell death up to 48 hours after siRNA treatment, in accordance with previous studies [22], [23]. However, we observed a large number of dying cells 72 hours after transfection with the hnRNP K siRNA; in contrast, cells treated with control siRNA did not show significant cell death. Activation of cell death pathways in Sy5y cells transfected with hnRNP K siRNA was verified by significant caspase-3 activity (n = 3, p<0.001) (Figure 4B), and increased TUNEL-positive cells (n = 6, p<0.0001) (Figure 4C), 72 hours post-transfection. The concentration of hnRNP K siRNA sufficient to activate caspase-3-mediated apoptosis at 72 hours was 100 pM (n = 3, p = 0.0001) (Figure 4D). Thus, down-regulation of hnRNP K activates caspase-3-mediated apoptosis similar to that observed in cells expressing expanded AUUCU repeats.

### hnRNP K over-expression rescues AUUCU–mediated apoptosis

Based on our data we postulated that an interaction of hnRNP K with the AUUCU repeat results in a loss of function of hnRNP K, leading to apoptotic cell death. We studied whether hnRNP K over-expression rescues cells from apoptosis induced by expanded AUUCU repeats. We established stably transfected Sy5y cell lines over-expressing hnRNP K, and then transiently transfected them with the plasmid shown in Figure 1C. Sy5y cells stably expressing a control plasmid in lieu of hnRNP K underwent massive cell death when expanded AUUCU repeats were expressed. In contrast, ∼50% over-expression of hnRNP K (Figure 4E) decreased the expanded AUUCU repeat-induced apoptosis by ∼30%, and this decrease in apoptosis is accompanied by reduced caspase-3 activity (n = 3, p<0.05) (Figure 4E). These data confirm our hypothesis that expanded AUUCU repeats activate apoptosis by suppressing hnRNP K function.

### PKCδ is accumulated within SCA10 and transgenic mitochondria

*In vivo* studies have shown that hnRNP K and PKCδ remain constitutively bound together within the cell [24]–[27]. Studies also showed that hnRNP K, when bound to nucleic acids, cannot be phosphorylated and cannot interact with PKCδ [25]. PKCδ has been implicated as an activator of apoptosis in many cell types, including neurons [28], [29]. Over-expression of PKCδ has been shown to activate apoptosis through a positive regulatory loop, in which caspase-3 activates PKCδ and activated PKCδ cleaves caspase-3 [30]. PKCδ over-expression results in its translocation to mitochondria, release of cytochrome c, and activation of caspase-3 [30]–[32]. Since binding of hnRNP K to the expanded AUUCU repeat is expected to reduce the formation of the hetero-dimeric complexes between hnRNP K and PKCδ, and mimic PKCδ over-expression, we investigated the ramifications of hnRNP K inactivation on sub-cellular localization of PKCδ in SCA10 fibroblasts and transgenic mouse expressing ∼500 AUUCU repeats in brain.

**Figure 5:**
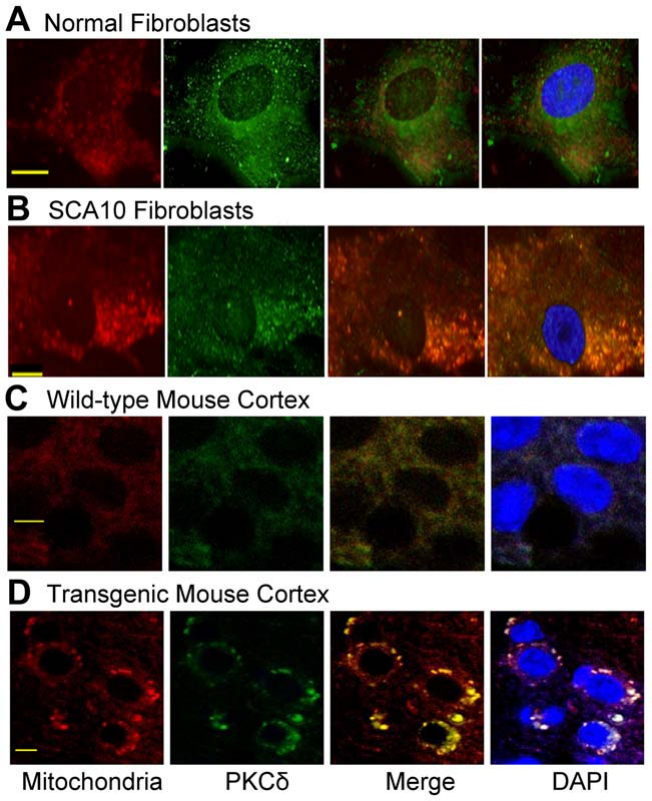
PKCδ is localized in mitochondria in SCA10 fibroblasts and transgenic mouse brain. (A) PKCδ (green) and mitochondria (red) in control normal fibroblasts. (B) PKCδ (green) in SCA10 fibroblast (punctate aggregates), primarily outside nuclei. Merge green (PKCδ with red (mitochondria) is seen as yellow/orange fluorescence. (C) PKCδ (green) and mitochondria (red) in the cortex of the control transgenic mouse brain expressing 12 ATTCT repeats. (D) PKCδ (green) and mitochondria (red) in SCA10 transgenic mouse cortex expressing ∼500 AUUCU repeats. Merge of green (PKCδ) and red (mitochondria) is shown as yellow/orange fluorescence in cortex of the SCA10 transgenic mice.

We first investigated whether cellular localization of PKCδ is altered in SCA10 cells. PKCδ was immunostained with green fluorescence and mitochondria were identified using mitotracker deep red 633. Immunostaining of the normal fibroblasts for PKCδ showed that PKCδ is present in the cytoplasm and the nucleus, but no significant PKCδ localization in mitochondria (Figure 5A). In contrast, PKCδ significantly overlaps with mitochondria in SCA10 fibroblasts as punctate staining around the nucleus, suggesting that a significant portion of PKCδ is translocated into the mitochondria (Figure 5B). To further verify that the interaction between hnRNP K and PKCδ is diminished in SCA10 fibroblasts, we immunoprecipitated hnRNP K from normal and SCA10 fibroblasts and analyzed the relative abundance of hnRNP K and PKCδ in the IP by Western blot analysis. The Western blot data show a significantly lesser amount of PKCδ in the IP from the SCA10 fibroblasts compared to normal fibroblast (Figure S4A). These data support our hypothesis that expanded AUUCU RNA interacts with hnRNP K and this binding results in the release of PKCδ, facilitating translocation of PKCδ to mitochondria in SCA10. To test that PKCδ is translocated to the mitochondria in SCA10 cells, we analyzed the mitochondrial protein fractions from SCA10 and control fibroblasts by Western blotting. Consistent with the immuno-histochemical data, the Western blot data showed elevated PKCδ level in SCA10 mitochondria (4B). Moreover, sagittal sections of transgenic mouse brain showed similar mitochondrial localization of PKCδ while negligible mitochondrial localization of PKCδ was seen in age-matched wild-type mice (Figure 5C and 5D). We also analyzed fibroblasts derived from patients with ataxia telangiectasia, and unlike SCA10 fibroblasts, these fibroblasts did not show presence of any detectable level of PKCδ in mitochondria (data not shown), suggesting the disease specificity of this mechanism in SCA10. These results suggest that PKCδ is translocated into the mitochondria of SCA10 cells.

### Down-regulation of hnRNP K or expression of AUUCU–RNA in normal fibroblasts result in translocation and accumulation of PKCδ in mitochondria

**Figure 6:**
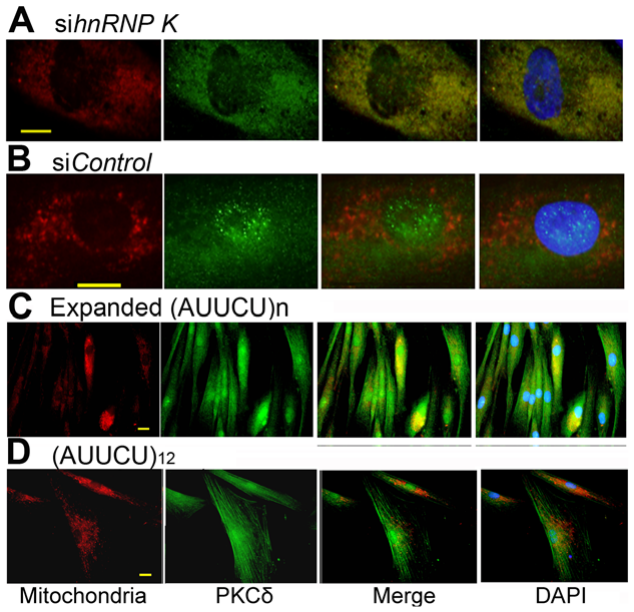
Targeted inactivation of *hnRNP K* in normal fibroblasts or expression of expanded AUUCU-RNA results in mitochondrial localization of PKCδ. (A) PKCδ (green) and mitochondria (red) in normal fibroblasts transfected with si*hnRNP K.* Merge of red and green fluorescence is seen as yellow/orange fluorescence. (B) PKCδ (green) and mitochondria (red) in normal fibroblast treated with si*Control*. Note very little yellow/orange fluorescence. (C) PKCδ (green) and mitochondria (red) in fibroblasts expressing ∼500 intronic AUUCU repeats. Co-localization of PKCδ and mitochondria is shown as yellow/orange fluorescence. (D) PKCδ (green) and mitochondria (red) in normal fibroblasts expressing 12 AUUCU repeats from an intron. Bar represents 10 mm in (A–D).

To test the hypothesis that the interaction of AUUCU RNA with hnRNP K leads to a loss function of hnRNP K, which then results in translocation of PKCδ into mitochondria, we transfected normal fibroblasts with hnRNP K siRNA and studied the cellular localization of endogenous PKCδ. When hnRNP K is downregulated, a majority of PKCδ was translocated to mitochondria and a negligible amount of PKCδ was detected outside mitochondria (Figure 6A). In normal fibroblasts, or in fibroblasts treated with control siRNA, most of the PKCδ was detected within cytoplasm and nuclei, with no detectable translocation to mitochondria (Figure 6B). Importantly, downregulation of hnRNP K in normal fibroblasts did not alter the steady state level of PKCδ (Figure S4C).

We also expressed ∼500 AUUCU repeats in primary human fibroblasts and studied the expression and cellular localization of PKCδ in these cells to investigate whether AUUCU RNA interferes with the expression and/or subcellular localization of PKCδ. We found that the expression of PKCδ remained unaltered in cells expressing expanded AUUCU repeats and the red fluorescence from mitochondria significantly overlaps with the green fluorescence from PKCδ in fibroblasts expressing ∼500 AUUCU repeats (Figure 6C and Figure S4D) or in those cells expressing ∼200 AUUCU repeats (Figure S5), suggesting that PKCδ translocates to mitochondria in response to the expression of expanded AUUCU repeats. In contrast, a negligible translocation of PKCδ was observed when 12 AUUCU repeats were expressed in fibroblasts (Figure 6D). Together, these data corroborate our hypothesis that the expanded AUUCU repeat interacts with hnRNP K, suppresses its function, resulting in mitochondrial translocation of PKCδ and activation of apoptosis.

### Targeted inactivation of *ATXN10* transcripts in SCA10 cells reduces mitochondrial accumulation of PKCδ

**Figure 7:**
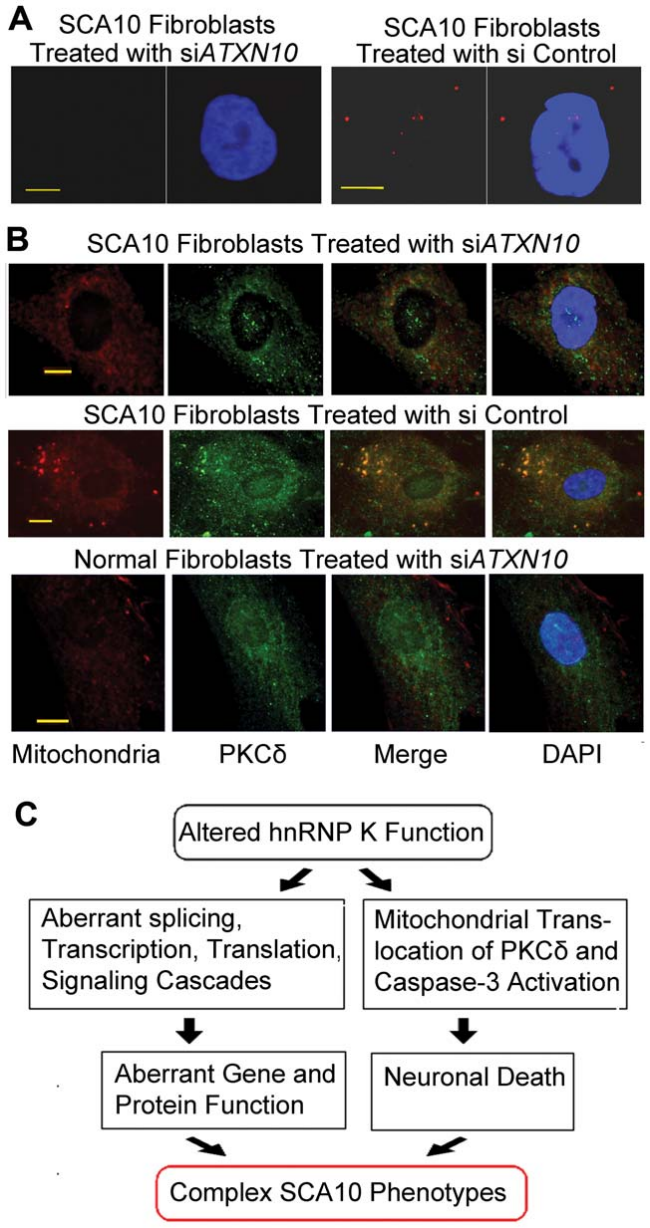
Down-regulation of *ATXN-10* in SCA10 fibroblasts decreases PKCδ localization in mitochondria. (A) *FISH* on SCA10 fibroblasts treated with si*ATXN10* (left) and with si*Control* (right). (B) Top panel: PKCδ (green) and mitochondria (red) in SCA10 fibroblasts 72 hours after treatment with si*ATXN10*. Center panel: PKCδ (green) and mitochondria (red) in SCA10 fibroblasts, 72 hours after treating with si*Control*, Bottom panel: PKCδ (green) and mitochondria (red) in human control fibroblasts. Bar represents 10 mm. (C) Schematic drawing describing a probable mechanism for the diverse phenotypes attributed with SCA10. The loss of hnRNP K function caused by the binding of expanded AUUCU-RNA can trigger multiple aberrant molecular signaling/processes in SCA10. Extensive neuronal loss may results from the activation of caspase-3 and cleavage of PKCδ triggering neuronal death. In parallel, either loss or diminished function of hnRNP K might result in aberrant splicing, transcription and translation of multiple genes contributing towards the complex disease phenotypes in SCA10.

To test whether mitochondrial localization of PKCδ can be decreased by reducing the mutant *ATXN10* transcript, we targeted the *ATXN10* transcript in SCA10 fibroblasts with two different ATXN10 siRNA and studied the cellular localization of PKCδ. This resulted in significant decrease in the number of both nuclear and cytoplasmic AUUCU RNA aggregates (Figure 7A; left panel), whereas control siRNA did not reduce the number of AUUCU RNA foci in SCA10 fibroblasts (Figure 7A; right panel). Treatment of SCA10 fibroblasts with ATXN10 siRNA substantially restored normal PKCδ subcellular localization, with decreased amount in mitochondria (Figure 7B; top panel). As expected, Control siRNA had no significant effects on the distribution or amount of PKCδ in SCA10 cells (Figure 7B; center panel). Treatment of normal fibroblasts with ATXN10 siRNA did not have any effect on PKCδ cellular localization (Figure 7B; bottom panel). We conclude that by disrupting the hnRNP K-AUUCU complexes, hnRNP K can re-establish its normal function within the cell, alleviating the pathogenic mechanisms leading to apoptosis. These findings support our hypothesis that expanded AUUCU repeats are toxic and are sufficient to trigger PKCδ translocation to mitochondria and apoptosis.

## Discussion

Multiple inherited human neurological disorders are now attributed to expansion of short tandem repeats either in coding or non-coding regions of genes [2], [7], [8]. Genetic and molecular analysis of these disorders have revealed that the repeat expansion can result in either a loss of function of the gene (Fragile-X syndrome and Friedreich's ataxia) or a gain of function of the encoded protein (SCA1, SCA2, SCA3, SCA6, SCA7, SCA17, Huntington's disease, DRPLA, and oculopharyngeal muscular dystrophy) [7], [8]. RNA-mediated pathogenesis is believed to play a critical role in several other repeat expansion disorders, including Myotonic Dystrophy Type 1 (DM1) and Type 2 (DM2), SCA8, SCA12, Huntington's disease like 2 (HDL2), and fragile X tremor ataxias syndrome (FXTAS) [7], [8]. However, the pathogenic mechanism of DM1, SCA8, SCA12, HDL2 and FXTAS, which are caused by trinucleotide repeat expansions, may also involve qualitative or quantitative alterations of the protein products of the respective genes or genes on the opposite strand [33]–[35]. In contrast, SCA10 is the only human disorder proven to be caused by an expansion of a pentanucleotide repeat. Like the DM2 CCTG tetranucleotide repeat, the SCA10 ATTCT repeat shows repeat-number polymorphism, which makes these non-trinucleotide repeat highly unlikely to encode protein sequences from either strand. Furthermore, we have shown that the intronic repeat expansion does not alter ATXN10 transcripts [17]. Thus, SCA10 is likely to be a disorder solely caused by RNA-based mechanism, unlike most disorders that are caused by trinucleotide repeat expansions.

In the present study we provide evidence that SCA10 pathogenesis results from a trans-dominant gain-of-function of AUUCU repeats. First, transcription of the mutant allele produces transcripts that form aggregates in the nucleus and cytoplasm of the SCA10 cells and in transgenic mouse brain. Second: the expanded AUUCU repeat complexes with hnRNP K, leading to the loss of function of hnRNP K. Third, expression of expanded AUUCU repeat results in the accumulation of PKCδ in the mitochondria and caspase-3 mediated activation of apoptosis. Fourth, diminished hnRNP K activity recapitulates these events caused by expanded AUUCU repeats. And finally, over-expression of hnRNP K, as well as down-regulation of transcripts of expanded ATTCT repeat, rescues cells from apoptosis caused by expanded AUUCU repeats. Based on these findings, we conclude that the AUUCU RNA binds to and inactivates hnRNP K, triggering caspase-3-mediated apoptosis via translocation of PKCδ to mitochondria. Previous reports suggest that the presence of PKCδ in the mitochondria results in decreased membrane potential, release of cytochrome C, and activation of caspase-3 [30]–[32], further supporting our conclusion. Moreover, caspase-3 activates PKCδ and activated PKCδ further activates caspase-3 [30], and proteolytically activated PKCδ down-regulates hnRNP K protein in a proteasome-dependent manner [36]. Hence, positive feedback loops involving hnRNP K, PKCδ and caspase-3 may enhance this pathogenic pathway in SCA10. Since apoptosis is considered to be a major mechanism of cell death in a variety of human neurodegenerative disorders [37], the novel pathway of apoptosis induced by the mutant *ATXN10* RNA is relevant to the neurodegenerative phenotype of SCA10. Our results provide strong evidence that this novel mechanism of trans-dominant RNA gain of function contributes to the pathogenic mechanism in SCA10.

The formation of aggregates may not necessarily be a required event for the mutant RNA to exert its toxicity. Binding of the soluble form of the mutant RNA to hnRNP K may be sufficient to cause the loss of function of hnRNP K with a release of PKCδ, and the aggregate formation could be a secondary phenomenon. We hypothesize that expanded AUUCU RNA pathologically binds to hnRNP K and prevents PKCδ from binding to the hnRNP K, mimicking over-expression of PKCδ within the cell. Previous studies have shown that hnRNP K is constitutively bound to PKCδ, but upon binding to nucleic acids, hnRNP K can no longer interact with PKCδ [25], [30]. Translocation of PKCδ to mitochondria in SCA10 cells, fibroblasts expressing expanded AUUCU repeat, and fibroblasts treated with hnRNP K siRNA argues for this mechanism. Studies have shown multiple apoptotic activators, including oxidative stress and over-expression of PKCδ, induce PKCδ translocation to the mitochondria, [32]. The mitochondrial translocation of PKCδ has been shown to cause an alteration in calcium signaling events and mediates the H_2_O_2_-mediated loss of membrane potential, release of cytochrome c, and activation of caspase-3 [38]. While it is possible that the expanded AUUCU repeat causes PKCδ translocation via other mechanisms, our data showing that over-expression of hnRNP K rescues AUUCU-mediated apoptosis argue for the mechanism mediated by a loss of function of hnRNP K.

Our present data do not rule out the possibility that additional proteins interact with the mutant *ATXN10* transcripts. Also, expression of hnRNP K is ubiquitous within the cell, and diminished hnRNP K could lead to altered regulation of transcription, splicing and cell signaling, which may account for the phenotypic variability in SCA10 as illustrated in Figure 7C. We are investigating these mechanisms. However, our current data convincingly show that the hnRNP K inactivation and PKCδ mitochondrial translocation are a key pathogenic pathway mediating the RNA gain of toxic function in SCA10.

## Materials and Methods

### Cell culture

SCA10 fibroblasts were isolated from skin biopsy from a Mexican-American SCA10 patient with ∼2000 repeats and a Brazilian SCA10 patient with ∼1000 repeats under signed informed consent approved by IRB at UTMB and the Ethics Committee at Federal University of Parana. These human fibroblasts Cells were cultured in MEM with Eagle-Earle salt and 2 mM L-glutamine containing 15% fetal bovine serum and antibiotic in 5% CO_2_ at 37°C in 75 cm^2^ flasks. Human neuroblastoma Sy5y cells were cultured at Ham's F12K medium with 2 mM L-glutamine adjusted to contain 1.5 g/L sodium bicarbonate, 15% horse serum, 2.5% fetal bovine serum in 5% CO_2_ at 37°C in 75 cm^2^ flasks.

### Construction of plasmids

The cytomegalo virus (CMV) promoter sequences in plasmid pCDNA3.1-hygro-lacZ (Invitrogen) were replaced with the *MfeI*/*BamHI* fragment of the human *α-enolase* promoter sequences (∼5.0 kb). The 2^nd^ intron of rabbit *β-globin* intron was cloned downstream of the enolase promoter and upstream of the *lacZ*. Expanded *ATTCT* repeats from the SCA10 hybrid cells [17] were PCR amplified with forward primer 5′-CCAAGGATGCAGGTGCCACAGCATCTC-3′ and reverse primer: 5′-ATATGCATCCAGCTTCTGATTACATGGACT-3′. A polylinker containing *Swa*I site was cloned into the *Mfe*I site within the *β-globin* intron. Subsequently, the DNA fragment containing the ATTCT duplex was cloned into the *Swa*I site within the intron. Presence of the expanded ATTCT sequences in the transgenic plasmid was confirmed by digesting the plasmid DNA with *Nhe*I and *Hind*III sites that flank the *Swa*I site, and by sequencing. Plasmids encoding the expanded ATTCT repeats were grown in E. coli SURE bacteria at 16°C to minimize the deletion of the repeat sequences [39]. The transgenic plasmid DNA containing the *LacZ* and ∼500 ATTCT repeats was digested with *MfeI* and *NaeI*, and digested DNA was electrophoresed on agarose gel. The ∼10 kb DNA fragment containing the transgene was purified from agarose gel using gel extraction kit (Qiagen). The cloned ATTCT repeats under enolase promoter contain 650 bp of upstream and 500 bp of downstream *ATXN10* sequence in addition to the ATTCT repeats. The control plasmids containing the same *ATXN10* flanking regions and 12 ATTCT repeats were PCR amplified from a normal subject. The cDNA clones of human hnRNP K was purchased from Open Biosystems, USA and cloned in-frame into pCGFP-C1 (BD Biosciences, USA). For construction of plasmid for stable expression of hnRNP K, the ORF of the human hnRNP K was PCR-amplified from the pool of human cDNA (Clonetech) with the following primer sets: forward: 5′-CTGATTGGTGTGCCCGTTTAATAA-3′ and reverse: 5′-CTCCTTCAGTTCTTCACTAGTC-3′. The 1507 bp PCR product was purified from agarose gel using gel extraction kit (Qiagen) and the blunt-ended PCR product was cloned into the *EcoR*V site in the mammalian expression vector pcDNA3.1-Hygro(+) (Invitrogen) to generate recombinant plasmid pcDNA-hnRNP K. The coding sequence of hnRNP K in recombinant plasmid pcDNA-hnRNP K was sequenced to verify the proper orientation and sequence integrity of hnRNP K.

### Development of the transgenic mouse line expressing 500 AUUCU repeats in brain

The transgene containing 500 ATTCT repeats within an intron (Illustrated in Figure 1C) was microinjected into the fertilized eggs and transplanted into the uterus of pseudo-pregnant surrogate mothers to obtain founder transgenic mice using standard procedure at the UTMB transgenic core facility. Presence of the transgene and the repeat in the transgenic founder mice were confirmed by both Southern blot as well as repeat primed PCR analyses. Animal experiments were performed under a protocol approved by IACUC at UTMB.

### Repeat-primed PCR and Southern blot analysis in transgenic mice

Mouse genomic DNA was collected from tail samples, and repeat-primed PCR and Southern blot analyses were performed as previously described [40].

### Identification of AUUCU–RNA–binding proteins

Plasmid pcDNA3.1 control as well as pcDNA-(ATTCT)n (n = 500) were first linearized with *Bam H*I and the linear plasmid was *in vitro* transcribed with T7 RNA polymerase (Promega). Biotinylated rCTP was mixed with other rNTPs during transcription to incorporate the biotin-labeled rCTP into (AUUCU)n RNA. Nuclear extracts from brain were made from a 2 month old B6 mouse using NE-PER Nuclear and Cytoplasmic Extract Reagent (Pierce) according to vendors specification. The total protein mixture was incubated with (AUUCU)n RNA at 4°C overnight, and the unbound proteins were washed by using RNA washing buffer (0.5% NP- 40, 100 mM NaCl, 50 mM Tris-HCl) four times. The proteins that remain bound to the magnetic beads after extensive washing were extracted by boiling the magnetic beads in 1X SDS-PAGE loading buffer. The extracted proteins were electrophoresed on 5–12% PAGE and proteins that appear as unique bands were excised, digested with trypsin and then analyzed by MALDITOF assay at Biomolecular Resource Facility Core at UTMB and the sequence was identified by searching the rodent protein database.

### Immuno-precipitation (IP) and RNA Co-IP

The SCA10 and control normal fibroblasts were harvested and washed with 1X PBS, lysed in 20 mM HEPES pH 7.4, 1 mM EDTA, 100 mM NaCl, 1% NP-40, Leupeptin (10 µg/ml), aprotinin (10 µg/ml), 20 mM β-glycero-phosphate, 20 mM NaF and 1 mM Na Vanadate. hnRNP K was immuno-precipitated using anti-hnRNP K antibody and the IP pellet was washed three times in the lysis buffer, and bound proteins were eluted in SDS-containing sample buffer and separated on SDS-PAGE. The membrane was first immunoblotted with anti-hnRNP K antibody and then with PKCδ antibody. To detect the intron 9 sequences of *ATXN10* in the IP pellet, total RNA was extracted from the IP pellets by phenol-chloroform extraction, and DNA contamination was removed with TURBO DNAse Kit (Ambion). The cDNA synthesis was carried out using 1 µg of total RNA using a RT-PCR kit (BD Biosciences). The cDNA aliquots were quantified and cDNA were used to detect the presence of intron 9 sequences of ATXN10 transcripts by PCR, using the forward primer 5′-AAGGATCAGAATCCCTGGAA-3 and the reverse primer 5′-TCATTCTGCCATCTGTTTTC-3′.

### The alternative splicing of β-tropomyosin

Splice isoforms of β-tropomyosin mRNA were analyzed using RT-PCR as previously described [20], [21] with a set of three primers; E5: 5′-GCCATGAAGGATGAGGAGAA-3′ (forward primer), E6a: 5′-CTGAGGTGGCCGAGAGGTAA-3′ (reverse primer to detect exon E6a) and E6b: 5′-TAAATGTGGGGACCTAGAGG-3′ (reverse primer to detect exon E6b).

### Solution binding assays of hnRNP K with (AUUCU)_15_ RNA

1 mg of the Streptavidin-conjugated Dynabeads (Invitrogen) was washed once with Solution A (100 mM NaOH, and 50 mM NaCl) and twice with Solution B (100 mM NaCl). The magnetic beads were next incubated in 50 µl of 2X incubation buffer (10 mM Tris, pH 7.5; 1 mM EDTA; 2 M NaCl,) for 15 minutes. 1000 pmoles (50 µl) of biotinylated RNA [either (AUUCU)_15_ or control RNA: (UUUCC)_3_(CCCUU)_3_(UUUUC)_3._] were added to the magnetic beads, incubated at room temperature for 1 hour, and washed twice with 1X incubation buffer. Five mg of purified hnRNP K were added to the magnetic bead-RNA mixture and incubated in a binding buffer (20 mM HEPES, pH 7.5; 10% glycerol; 1 mM DTT; 0.1 mM EDTA) at 4°C for overnight. The supernatant was discarded and the magnetic beads were resuspended in binding buffer (25 mM Tris, pH 7.5; 0.1 mM EDTA; 10 mM NaCl). RNA-bound hnRNP K was next sequentially eluted with buffers containing increasing NaCl concentrations. The eluted hnRNP K protein fractions were analyzed on PAGE by Coomasie staining and Western blotting.

### Antibodies and western blots

Mouse monoclonal anti-hnRNP K was obtained from Acris Antibodies GmbH. Monoclonal anti-PKCδ (G-9) and Cytochrome C Oxidase (COXII) antibodies were purchased from Santa-Cruz Biotechnology. The Western blotting experiments were done according to standard procedure and the target proteins were detected using ECL Western kit (Amersham). Expression levels of β-actin (Abcam) were used as controls for protein loading.

### TUNEL assay

Cells were grown in chamber slides overnight prior to TUNEL assay. TUNEL assay was performed according to vendor instructions (Roche). Student's t-test was used to calculate statistical significance.

### Caspase-3 assay

Caspase-3 assay was performed according to instructions supplied by vendor (Calbiochem). P values were calculated using student's t-test.

### Transfection

Sy5y cells were transfected with plasmids or siRNA by Lipofectamine 2000 reagent (Invitrogen). For the targeted inactivation of *hnRNP K*, the On-target-Plus siRNA duplexes were purchased from Dharmacon. The control non-targeting siRNA from Dharmacon was used as a negative control for all siRNA experiments. Approximately 100 and 200 nmoles of the siRNA pools were used for the targeted down-regulation of hnRNP K, and different assays were applied 72 hours after the transfection. Fibroblasts were transfected using the human dermal fibroblast nucleofector kit for electroporation (Amaxa Corporation). Plasmids expressing either 12 or 500 ATTCT repeats were transfected at 3 µg according to kit instructions, and siRNA (both hnRNP K and ataxin 10) was transfected according to kit instructions. For stable over-expression of hnRNP K in Sy5y cells, pcDNA-hnRNP K was digested with *Mfe*I and the linear plasmid DNA was tranefected into Sy5y cells using Lipofectamin 2000 reagent (Invitrogen), and the stable clones were selected with hygromycin (300 mg/ml). Total protein was isolated from the stably transfected cells and the hnRNP K expression level was analyzed by Western blotting with the anti-hnRNP K antibody.

### Fluorescent *In* Situ Hybridization (FISH)

RNA foci were detected using a Cy3-labeled (AGAAU)_10_ RNA riboprobe. Slides were pre-hybridized at 65°C in RNA hybridization buffer for 1.5 hours. Slides were then hybridized overnight in 250 ng (AGAAU)_10_/1 ml hybridization solution at 45°C. Slides were rinsed with PBS three times and then extensively washed 4 times 5 minutes each to remove all non-specific binding probes. Slides were then mounted with DAPI mounting medium.

### FISH and Immunohistochemistry

Transgenic mice anesthetized with Avertin were perfused through the aorta, first rinsing for 15 minutes with PBS and then 60 ml of fresh 4% Paraformaldehyde (PFA) in DEPC water. The brain was carefully removed and stored in 4% PFA at 4°C with gentle agitation overnight. Brain tissue was then placed in 30% sucrose overnight. Mouse brains were fixed in paraffin and sectioned sagittally. RNA foci were stained using a Cy3-labeled (AGAAU)_10_ riboprobe. First, paraffin was removed from the brain sections and slides were dehydrated with 70%, 95% and 100% Ethanol in DEPC water, and washed using DEPC PBS. Following *FISH*, hnRNP K was immunodetected. Sections were blocked with DAKO antibody blocking solution (serum-free) and later double stained with anti-hnRNP K 1∶1000 in DAKO antibody diluent. Goat anti-mouse 488 was used to identify hnRNP K and slides were visualized using a Hamamatsu Camera Controller using DP controller software in histopathology lab at UTMB.

### Translocation of PKCδ into mitochondria

Fibroblasts were transfected with plasmids pcDNA-(ATTCT)_12_, pcDNA-(ATTCT)_500_, hnRNP K siRNA or control siRNA through electroporation. Transfections were conducted in chamber slides. Thirty-six hours after repeat transfection and 72 hours after RNAi transfection, the cells were treated with mitotracker deep red 633 (Invitrogen) at a concentration of 250 nM in cell culture medium. Cells were incubated at 37°C for 30 minutes. After washing the cells three times with PBS, cells were then fixed with 4% PFA for 30 minutes at room temperature. Cells were washed 3 times with PBS and stored in 70% Ethanol for up to 24 hours. Cells were blocked with DAKO antibody blocking solution (serum-free) and later double stained with anti-PKCδ 1∶500 in DAKO antibody diluent. Goat anti-mouse 488 was used to identify PKCδ. Fluorescent photomicrographs were taken using a Hamamatsu Camera Controller using DP controller software in the histopathology core lab at UTMB.

The sagittal section of the transgenic brain was processed according to the procedure described above and immuno-stained with anti-PKCδ and Cox II antibodies to detect mitochnodria and PKCδ respectively. The cytoplasmic, nuclear and mitochondrial protein fractions from normal and SCA10 cells were isolated using the mitochondria isolation kit and Sub-cellular Protein Fractionation kits (Thermo Scientific-Pierce). The isolated proteins were analyzed by Western blotting using anti-PKCδ antibody.

## Supporting Information

Figure S1 Mutant *ATXN10* transcripts are deposited as insoluble aggregates. (A) Aggregates in SCA10 fibroblasts encoding ∼1000 CUG repeats: *FISH* showing the nuclear and cytoplasmic AUUCU-RNA aggregates (arrow) in SCA10 fibroblasts encoding ∼1000 ATTCT repeats. (B) Aggregates in normal human fibroblasts ectopically expressing transcripts containing expanded AUUCU repeats: *FISH* showing SCA10-like aggregates (arrow) in normal fibroblasts transfected with plasmid encoding ∼500 ATTCT repeats as schematically shown in Figure 1C. (C) Aggregates in transgenic mouse brain expressing ∼500 AUUCU repeats: Upper panel: *FISH* showing nuclear, perinuclear and cytoplasmic AUUCU-RNA foci or aggregates in a 3 month old transgenic mouse brain expressing ∼500 intronic AUUCU repeats from the transgene described in Figure 1C. Lower panel: Control mouse brain expressing 12 AUUCU repeats.
(1.12 MB TIF)

Figure S2 Expanded AUUCU repeats induces apoptosis in PC12 cells. (A) TUNEL assay on PC12 cells expressing ∼500 and 12 intronic AUUCU repeats. (B) Percentage of cells undergoing apoptosis when 12 and ∼500 intronic AUUCU repeats are expressed in PC12 cells (n = 6; p<0.00032). (C) Bar diagram showing Caspase-3 activities as relative fluorescent units (RFU) when PC12 cells express 12 and ∼500 AUUCU intronic repeats (*n = 4, p<0.0002).
(0.91 MB TIF)

Figure S3 hnRNPK co-localizes with the expanded AUUCU-RNA in the transgenic cortex. *FISH* showing co-localization of hnRNP K (green) with AUUCU RNA (red) in sagittal sections of the SCA10 transgenic mouse cortex (3 month old) expressing ∼500 intronic AUUCU repeats expressing from the transgene as described in Figure 1C. Bars represent 10 mm.
(0.88 MB TIF)

Figure S4 Interaction and levels of hnRNP K and PKCδ in SCA10 cell models. (A) Interaction of hnRNP K with PKCδ is diminished in SCA10 cells: The Western blot showing PKCδ and hnRNP K levels in the IP from the SCA10 cells expressing ∼2000 AUUCU repeats and normal fibroblasts expressing 12 AUUCU repeats. (B) PKCδ levels in the mitochondrial protein fractions in normal and SCA10 fibroblasts. The Western blot showing PKCδ levels in normal (lane 1 and 2) and SCA10 mitochondria (Lane 3 and 4): Cytochrome C Oxidase II (COX II) was used as loading control of the mitochondrial protein fractions. (C) Down-regulation of hnRNP K does not alter the steady state level of PKCδ. Western blot showing PKCδ levels in normal fibroblasts (Lane 1) and fibroblasts treated with 100 pmoles (Lane 2) and 200 pmoles (Lane 3) of hnRNP K-siRNA. (D) Ectopic expression of AUUCU repeats does not alter the steady state level of endogenous PKCδ. Western blot showing the steady state level of PKCδ in normal human fibroblasts (Lane 1) and in SCA10 fibroblasts expressing ∼500 AUUCU repeats (Lane 2).
(0.12 MB TIF)

Figure S5 Expression of ∼200 AUUCU repeats in normal fibroblasts results in massive mitochondrial localization of PKCδ. *FISH* showing PKCδ (green) and mitochondria (red) in normal fibroblasts transfected with plasmid encoding ∼200 ATTCT repeats: Merge of red and green fluorescence from mitochondria and PKCδ respectively is seen as yellow/orange fluorescence.
(0.98 MB TIF)
