# Supplementary figures and images for: Inactivation of hnRNP K by Expanded Intronic AUUCU Repeat Induces Apoptosis Via Translocation of PKCδ to Mitochondria in Spinocerebellar Ataxia 10
Source: PLoS Genet. 2010 Jun 10;6(6):e1000984. doi: 10.1371/journal.pgen.1000984 (PMC2883596; doi:10.1371/journal.pgen.1000984)

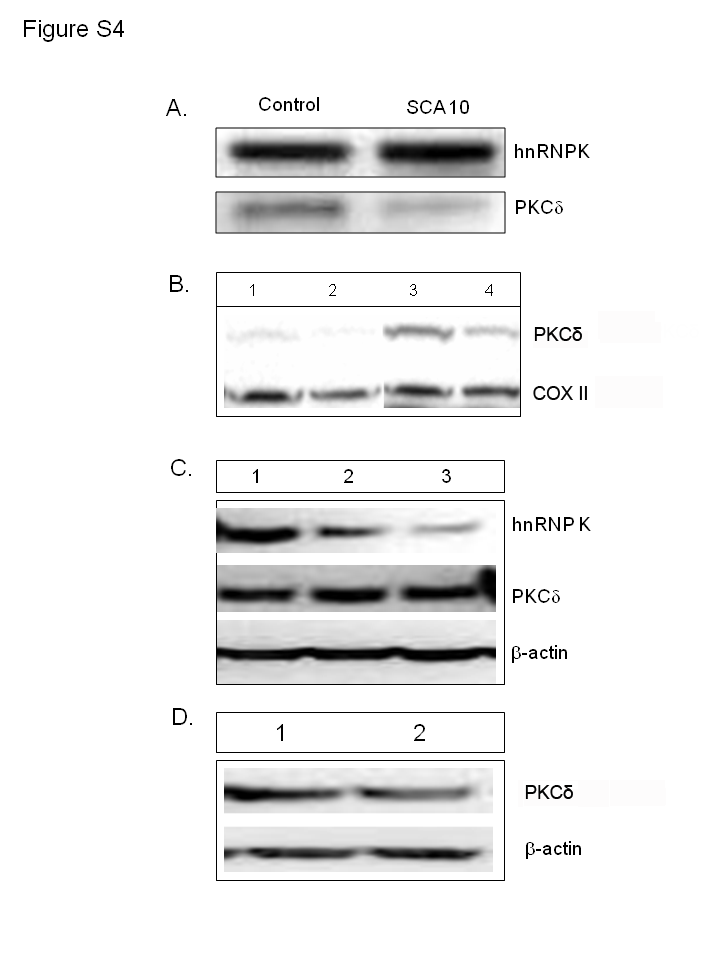

Supplement: Figure S4 — Interaction and levels of hnRNP K and PKCδ in SCA10 cell models. (A) Interaction of hnRNP K with PKCδ is diminished in SCA10 cells: The Western blot showing PKCδ and hnRNP K levels in the IP from the SCA10 cells expressing ∼2000 AUUCU repeats and normal fibroblasts expressing 12 AUUCU repeats. (B) PKCδ levels in the mitochondrial protein fractions in normal and SCA10 fibroblasts. The Western blot showing PKCδ levels in normal (lane 1 and 2) and SCA10 mitochondria (Lane 3 and 4): Cytochrome C Oxidase II (COX II) was used as loading control of the mitochondrial protein fractions. (C) Down-regulation of hnRNP K does not alter the steady state level of PKCδ. Western blot showing PKCδ levels in normal fibroblasts (Lane 1) and fibroblasts treated with 100 pmoles (Lane 2) and 200 pmoles (Lane 3) of hnRNP K-siRNA. (D) Ectopic expression of AUUCU repeats does not alter the steady state level of endogenous PKCδ. Western blot showing the steady state level of PKCδ in normal human fibroblasts (Lane 1) and in SCA10 fibroblasts expressing ∼500 AUUCU repeats (Lane 2). (0.12 MB TIF) [file pgen.1000984.s004.tif]
